# Viromics on Honey-Baited FTA Cards as a New Tool for the Detection of Circulating Viruses in Mosquitoes

**DOI:** 10.3390/v12030274

**Published:** 2020-02-29

**Authors:** Lotty Birnberg, Sarah Temmam, Carles Aranda, Florencia Correa-Fiz, Sandra Talavera, Thomas Bigot, Marc Eloit, Núria Busquets

**Affiliations:** 1 Centre de Recerca en Sanitat Animal (CReSA), Institut de recerca en Tecnologies Agroalimentaries (IRTA), 08193 Barcelona, Spain; lotty.birnberg@irta.cat (L.B.); carles.aranda@irta.cat (C.A.); flor.correa@irta.cat (F.C.-F.); sandra.talavera@irta.cat (S.T.); 2 Institut Pasteur, Pathogen Discovery Laboratory, 75015 Paris, France; sarah.temmam@pasteur.fr (S.T.); thomas.bigot@pasteur.fr (T.B.); marc.eliot@pasteur.fr (M.E.); 3 Servei de Control de Mosquits del Consell Comarcal del Baix Llobregat, 08820 Barcelona, Spain; 4 Institut Pasteur – Bioinformatics and Biostatistics Hub—Computational Biology department, Institut Pasteur, USR 3756 CNRS—75015 Paris, France; 5 National Veterinary School of Alfort, Paris-Est University, 94704 CEDEX, Maisons-Alfort, France

**Keywords:** FTA cards, NGS, insect specific virus, saliva, *Alphamesonivirus*, *Quaranjavirus*, unclassified *Bunyavirales*

## Abstract

Worldwide, emerging and re-emerging infectious diseases (EIDs) are a major burden on public and animal health. Arthropod vectors, with mosquitoes being the main contributors of global disease, transmit more than 70% of the recognized EIDs. To assess new alternatives for arthropod-borne viral diseases surveillance, and for the detection of new viruses, honey-baited Flinders Technology Associates (FTA) cards were used as sugar bait in mosquito traps during entomological surveys at the Llobregat River Delta (Catalonia, Spain). Next generation sequencing (NGS) metagenomics analysis was applied on honey-baited FTA cards, which had been exposed to field-captured mosquitoes to characterize their associated virome. Arthropod- and plant-infecting viruses governed the virome profile on FTA cards. Twelve near-complete viral genomes were successfully obtained, suggesting good quality preservation of viral RNAs. Mosquito pools linked to the FTA cards were screened for the detection of mosquito-associated viruses by specific RT-PCRs to confirm the presence of these viruses. The circulation of viruses related to *Alphamesonivirus*, *Quaranjavirus* and unclassified *Bunyavirales* was detected in mosquitoes, and phylogenetic analyses revealed their similarities to viruses previously reported in other continents. To the best our knowledge, our findings constitute the first distribution record of these viruses in European mosquitoes and the first hint of insect-specific viruses in mosquitoes’ saliva in field conditions, demonstrating the feasibility of this approach to monitor the transmissible fraction of the mosquitoes’ virome. In conclusion, this pilot viromics study on honey-baited FTA cards was shown to be a valid approach for the detection of viruses circulating in mosquitoes, thereby setting up an alternative tool for arbovirus surveillance and control programs.

## 1. Introduction

Worldwide, two-thirds of all recognized emerging and re-emerging infectious diseases (EIDs) are of viral origin [1], with arthropod-borne viruses (arboviruses) being the causative agents of more than 30% of them [2]. Arboviruses circulate naturally between their vertebrate hosts and vectors. Nearly 135 arboviruses are known to infect humans, posing a significant threat to public health [3]. Globalization, together with anthropic activities and climate change, has facilitated the dispersal of pathogenic agents (arboviruses included), their hosts and vectors, extending the risk to more and newer areas [4,5]. Since the increased incidence of dengue [6], Zika [7,8], chikungunya [9,10] and West Nile viruses [11], there is a growing interest in understanding the viral diversity harbored by arthropod vectors, and a rising necessity to develop more effective surveillance and monitoring tools for circulating viruses.

Traditionally, for active surveillance and control purposes, samples from entomological surveys and/or from sentinel animals are subjected to laboratorial analyses to evidence arbovirus circulation. Despite these methodologies being considered the “gold standards”, many issues must be considered. For instance, in entomological surveys, specialized personnel are required to capture and classify specimens, and a cold chain must be maintained to prevent virus degradation until molecular processing [12,13]. Due to the low prevalence of infected individuals between inter-epidemic periods, large numbers of mosquitoes have to be analyzed to detect a virus [13]. When using sentinel animals, besides the necessary logistics, ethical considerations have to be taken into account, as the physical integrity of the animals, as well as that of the personnel, should be warranted [14]. Likewise, customary laboratorial techniques for virus detection present some limitations, for example in serological diagnosis, closely related viruses may produce cross-reactions [14], while PCR-based techniques target only those viral lineages that are already known, thereby underestimating the diversity of the sample while overlooking undescribed viruses that could potentially be pathogenic [15].

Since gold-standard strategies are time-consuming, logistically complex and potentially hazardous, honey-baited Flinders Technology Associates (FTA) cards have been used as an alternative tool for arbovirus surveillance as they inactivate pathogens and preserve nucleic acids on contact, thereby simplifying the labor [12,13,16]. In previous field trials, honey-soaked FTA cards have been used in combination with molecular techniques to detect several arboviruses, such as Ross River virus (RRV), Barmah Forest virus (BFV) [13,16,17,18] and West Nile virus strain Kunjin (WNVKUN) [13,17] in Australia, and Usutu virus (USUV) in Switzerland [19]. Moreover, while virological surveillance in mosquitoes is based mainly upon virus detection in entire mosquitoes, indicating that they might be infected, the detection of viruses expectorated within the saliva during sugar feeding and deposited directly on the FTA cards may identify infectious mosquitoes [18].

To overcome the detection bias of molecular-based techniques, deep sequencing technologies have been proven as a valid approach to detect, characterize and discover unknown or uncultured viruses within biological or environmental samples [20,21,22,23]. Recently, by high throughput sequencing, diverse and widely distributed novel non-taxonomic groups of RNA viruses that naturally infect insects have been discovered in mosquitoes. Between 2007 and 2017, 187 novel mosquito-associated viruses have been reported and classified within 25 families [24]; some of them commonly grouped with human/animal arboviral pathogens or plant viruses. The capacity to detect untargeted viruses enables metagenomics to act as a new and powerful approach to enhancing arbovirus surveillance programs [25]. 

To the best of our knowledge, for the first time, next generation sequencing (NGS) on honey-impregnated FTA cards used as sugar bait during entomological surveys has been tested as a new approach for the detection of viruses circulating in mosquitoes. Viromics results on FTA cards were confirmed by the detection of mosquito-associated viruses in field-captured mosquitoes. Additionally, near-complete viral genomes were obtained. Herein, we show that insect-specific viruses (ISVs) can be detected in saliva from field-captured mosquitoes and report some ISVs previously identified in other continents, as first-distribution records in European mosquitoes.

## 2. Materials and Methods 

### 2.1. Study Area and Sampling Strategy

The present study was conducted at the Llobregat River Delta, North-Eastern Spain. In this Delta, densely populated areas coexist with natural habitats that serve as a strategic stopover on the route of migratory birds between Europe and Africa. For this reason, this area is considered to be of particular epidemiological interest and is targeted for arbovirus surveillance. In fact, sampling locations were chosen based on previous evidence of arbovirus circulation [26], and in places where the Servei de Control de Mosquits del Baix Llobregat performs regular mosquito monitoring and control activities. Peri-urban and rural biotopes within this area were sampled to provide variability and increase the probability of virus detection. 

Every fortnight, from May to November 2015, host-seeking female mosquitoes were captured using CO_2_-baited EVS Mosquito Traps (Bioquip, Compton, CA, USA). Inside the collection bag of some traps, one honey-soaked Classic FTA^TM^ card (Whatman^TM^, GE Healthcare UK limited, Buckinghamshire, UK) [15] was placed as a sugar-bait for the captured specimens. Only the honey-impregnated area of the card was left exposed to allow specimens feed on it while in the trap [27]. At each location, traps with and without honey-baited FTA cards were placed indiscriminately and kept operational from the early evening to the next morning (approximately 18 h). After sampling periods, FTA cards were removed, covered with Parafilm^®^ (Bemis, Neenah, WI, USA) and coded according to location and sampling date. Only captured female mosquitoes were morphologically classified [28] and up to 30 individuals were pooled according to species, location and sampling date. A few non-culicid dipterans were also captured but not classified. The number of specimens with blue abdomens was recorded per species as evidence of feeding on the FTA cards. A cold chain was maintained through specimen transportation and handling to avoid RNA degradation [27]. FTA cards and specimens were preserved at −80 °C until molecular analysis. 

### 2.2. RNA Extraction from FTA cards for NGS Analysis

Pre-extraction, frozen FTA cards were thawed at 4 °C, homogenized with 500 µL of cold sterile PBS by vortex and squeezed with a sterile pestle to extract its content. Total RNA was obtained from individual FTA cards (13 peri-urban and 23 rural) using the RNeasy Mini Kit (QIAGEN GmbH, Hilden, Germany) according to the manufacturer’s instructions. Extracted RNA was eluted in 50 µL of RNase-free water. A unique RNA sample per biotope was generated by pooling 15 µL of all the corresponding extracts of the given area.

### 2.3. Library Preparation, Sequencing and Bioinformatics Analysis

RNA samples were sequenced and analyzed as previously described with slight modifications [29]. Briefly, to obtain complementary DNA (cDNA), RNA samples were retro-transcribed using random hexamers and the SuperScript IV reverse transcriptase (Invitrogen, Vilnius, Lithuania). Random amplification of cDNAs was performed using the multiple displacement amplification (MDA) protocol with phi29 polymerase and random hexamers [30]. Libraries were sequenced at a depth of 60 to 80 million reads on an Illumina HiSeq2000 platform in a 150-base pairs (bp) single read format, outsourced to DNAvision Company (Charleroi, Belgium). 

Raw reads were processed with an in-house bioinformatics pipeline as previously described [31]. Summarizing, it comprised quality check and trimming based on AlienTrimmer package [32] (Phred quality score cutoff = 80, min % of correctly called nt = 20) followed by read normalization using BBnorm program (https://jgi.doe.gov/data-and-tools/bbtools) (cut-off parameter of 100). De novo assemblies were performed using Megahit tool [33] (minimum contig length = 100 nt). For further ORF prediction ((https://figshare.com/articles/translateReads_py/7588592), minimum aa length = 15), a Diamond-based similarity search (v0.9.22.123) against the protein Reference Viral database (RVDB-prot 16.0 [34]) was conducted. Validation of viral taxonomic assignations was accomplished by a first Diamond-based search against the whole protein NCBI/nr database (1 November 2019 version) and a final search against the whole NCBI/nt nucleotide database (15 August 2019 version) to discard any putative non-viral intronic sequences that would, by chance, present a significant similarity with a viral protein. The pipeline used performs a protein blast for each viral contig and singleton, and then analyzes the taxonomic classification for all the co-best hits (meaning all the hits that have the same score). If all the hits were assigned to the same species, this species was reported as the closest hit. If the assembly had two or more different species or genera classifications, the last common ancestor was reported—genus or family, respectively. For low-level identities, taxonomic assignations were suggestive of putative new viral sequences. The quantification of abundance of each viral taxon was obtained by summing the length (in nucleotides) of all sequences being associated to this taxon, weighted by the *k-mer* coverage of each contig.

### 2.4. Primers Design and Virus Detection by Specific RT-PCRs

To confirm that viruses reported by metagenomics on FTA cards come solely from the captured specimens and not from the honey-bait, sequences assigned to mosquito-associated viruses were extracted. Among these, four viruses, with at least one assembly longer than 1000 nucleotides (nts) and with an identity higher than 90% were selected. Then, primers were designed from the extracted sequences of each chosen virus and conventional virus-specific reverse transcription polymerase chain reactions (RT-PCR) were set up. Viral RNA from mosquito pools and honey-baited FTA cards, which had not been exposed to mosquitoes, were then extracted using NucleoSpin^®^ RNA Virus kit (Macherey–Nagel, Düren, Germany) following the manufacturer’s instructions. Using the OneStep RT-PCR kit (QIAGEN GmbH, Hilden, Germany), all the above-mentioned samples were screened for the detection of Alphamesonivirus 1, *Bunyaviridae* environmental sample, Dezidougou virus and Wuhan mosquito virus 7, adjusting the annealing temperatures to each set of primers (Table 1). As positive amplification controls, Dezidougou virus isolate and Alphamesonivirus cDNA were used (kindly provided, respectively, by Scott Weaver from the World Reference Centre for Emerging Viruses and Arboviruses at University of Texas Medical Branch (WRCEVA–UTMB), and Patricia Gil and Serafín Gutiérrez from Centre de Coopération Internationale en Recherche Agronomique pour le Développement (CIRAD) at Montpellier). Meanwhile, for other viruses, since viral isolates were not available, extracted RNA from the FTAs that had been subjected to metagenomics were used as positive amplification controls. Amplification products were visualized in 2% agarose gels with ethidium bromide (0.1 µg/mL) staining. 

### 2.5. Sequencing and Phylogenetic Analyses

Virus-specific RT-PCR products were purified using the QIAquick^®^ Gel Extraction Kit (QIAGEN GmbH, Hilden, Germany) and Sanger sequenced in both directions using the BigDye^®^ Terminator v3.1 cycle Sequencing Kit (Life Technologies Corporation, Austin, TX, USA). At the Servei de Genomica i Bioinformatica at the Universitat Autonoma de Barcelona (SGB-UAB), amplicons were purified with the BigDye X Terminator kit (Applied Biosystems™, Waltham, MA, USA) and subjected to capillary electrophoresis in the Genetic Analyzer 3130xl (Applied Biosystems™, USA). Viral sequences were aligned using BioEdit Sequence Alignment Editor [35] and the identity of each virus was confirmed by comparing them to GenBank’s reference database using the nucleotide Basic Local Alignment Search Tool (BLASTn) algorithm. At least one viral sequence per geographic region and a year that exhibited high similarities in the BLAST analysis to our subject sequences was used to infer the phylogenetic relationship of each studied virus. Viral sequences were then pairwise aligned using ClustalW algorithm in the Molecular Evolutionary Genetics Analysis program version X (MEGAX) [36]. In the same program, phylogenetic trees were constructed using the Maximum Likelihood (ML) method. Based on the Bayesian information criterion (BIC) score [36,37] the best models were applied. Tamura-Nei (TN93+G) with gamma distributions showed to be the best fit for Alphamesonivirus/CAT and Wuhan mosquito/CAT viruses, and Hasegawa-Kishino-Yano (HKY+G) [38] with gamma distributions the best fit for *Culex* bunyavirus/CAT virus. In both cases, a 1000 replicate bootstrap was used. 

### 2.6. Nucleotide Sequences Accession Numbers

The raw sequencing datasets for both batches of honey-baited FTA cards are available in the NCBI Sequence Read Archive (SRA) repository under the BioProject ID: PRJNA604676 (www.ncbi.nlm.nih.gov/biosample/13978317 and www.ncbi.nlm.nih.gov/biosample/13978318). All the viral genomes for which the complete CDS were obtained were deposited in the GenBank archive under the accession numbers: MT096515-MT096531. Sequences corresponding to the viruses detected in mosquito pools from the Llobregat River Delta are available under the accession numbers: MT063093-MT063099. 

## 3. Results and Discussion

After sampling periods at the Llobregat River Delta, 1080 female mosquitoes were collected and classified into five species: *Aedes albopictus* (*n* = 20; 10 pools), *Coquillettidia richiardii* (*n* = 11; 5 pools), *Culex pipiens* (*n* = 755; 53 pools), *Aedes caspius* (*n* = 294; 24 pools) and *Aedes detritus* (*n* = 2; 1 pool) (Table S1). A total of 38 honey-baited FTA cards were recovered; 36 linked to mosquito captures and two from traps with no captures. Batches of 13 FTA cards from peri-urban and of 23 FTA cards from rural biotopes linked to mosquito captures constituted two independent samples for metagenomics analysis. Visual inspections depicted blue abdomens in 21% and 39% of the captured mosquitoes, respectively, for peri-urban and rural biotopes, confirming that they had fed on the FTA cards while in the trap. No evidence of blue dye was observed in *Ae. detritus* (Table S1). 

### 3.1. Outputs on NGS on Honey-Baited FTA Cards

Next generation sequencing (NGS) on honey-baited FTA cards generated 61,362,209 and 80,631,320 of raw reads for rural and peri-urban biotopes, respectively. After filtering steps, 56,424,764 and 76,884,845 reads of 150 bases were assembled to produce 431,179 and 100,469 contigs respectively for rural and peri-urban datasets. Depurated reads also generated 3,128,224 and 846,017 singletons in each case. 

### 3.2. Virome Composition on Honey-Baited FTA Cards During Entomological Surveys

Taxonomic assignations of the viral sequences obtained by high throughput sequencing on honey-baited FTA cards revealed that more than 95% corresponded to RNA viruses. *Picornavirales*, *Nidovirales* and *Tymovirales* were the most represented single-stranded positive sense RNA (ssRNA+) viral orders; and *Bunyavirales* the most abundant single-stranded negative sense RNA (ssRNA-) order. Double-stranded RNA (dsRNA) viral families *Partitiviridae* and *Totiviridae* were also dominant. DNA and unclassified viruses comprised the remaining 5% of the viral diversity herein reported (Table S2). In agreement with previous virome studies, most of the taxa derived from honey-baited FTA cards have been identified in various invertebrates [39] and associated to mosquitoes [40]. Additionally, mosquito-specific viruses detected in FTA cards (Table 2) have been described as part of the viral communities harbored by several mosquito species in different geographic regions [41,42,43,44,45,46,47,48]. 

**Figure 1:**
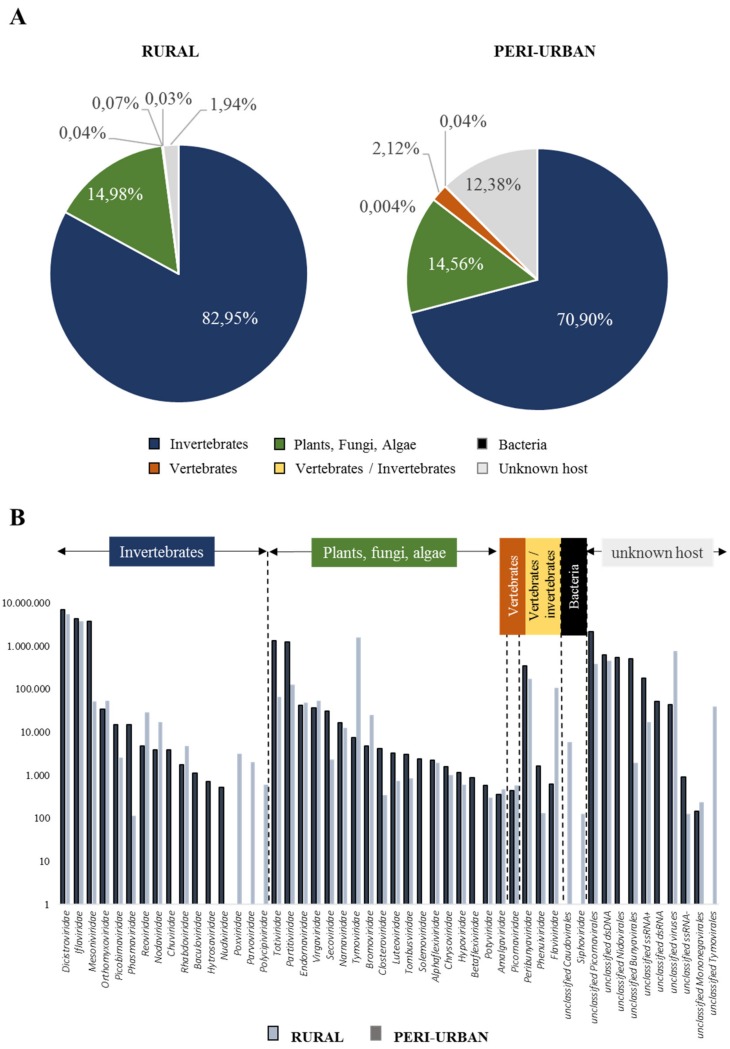
Overview of viral composition of honey-baited FTA cards. (**A**) Shows the proportion of viral reads classified by host type. Proportions of bacteria and vertebrate/invertebrate are too small to be seen in the figure. (**B**) Abundance in nucleotides of each viral family estimated by summing sequence length in nucleotides weighted by the k-mer coverage of each contig.

In the present study, taxonomic profiling revealed the prevalence of invertebrate-associated viruses (Figure 1A) with *Dicistroviridae*, *Iflaviridae* and *Mesoniviridae* being the most abundant families (Figure 1B). Sequences herein designated as *Dicistroviridae* and *Iflaviridae* (order *Picornavirales*) were mostly related to hymenopterans, in particular to the honeybee *Apis mellifera*. Since we could not sequence the honey used to impregnate the FTA cards as sugar bait, we cannot discard the possibility that these sequences might have come from it. However, recent virome studies have described these two families as the most abundant in culicid mosquitoes from the Yunnan province in China, and Zambezi province in Mozambique [49,50]. The additional description of honeybee-infecting virus *Rhopalosiphum padi* virus (*Dicistroviridae*, genus *Cripavirus*) in mosquito species from Hubei, China [51] and in *Culex* mosquitoes from California [41], together with the assembly of sequences linked to chronic bee paralysis virus (CBPV) (unclassified ssRNA+ virus) and *Apis mellifera* filamentous virus (dsDNA *Hytrosaviridae* family) from French *Anopheles maculipennis* [52] and from *Culex* mosquitoes from California [41] respectively, suggested that these viral families could be associated to mosquitoes as well. In addition, due to the low genetic identity of these viruses with their closest honeybee counterpart, the scarcity of mosquito-based sequences available in public databases, and the continuous discovery of new picorna-like viruses in insects [41,45,50,53,54], might suggest that we are dealing with novel mosquito picorna-like viruses. Based on the abovementioned findings, captured mosquitoes that fed on the FTA cards could have been the source of the identified viruses. 

Besides invertebrate-related viruses, it was not surprising to find viral families usually detected in plants, fungi and algae (e.g., *Tymoviridae*, *Totiviridae*, *Partitiviridae*, *Endornaviridae* or *Virgaviridae*) as part of the viral diversity associated to honey-baited FTA cards (Figure 1B). Since, in *Culex* mosquitoes, sequences related to *Totiviridae*-like viruses have been found in Guadeloupe [46], Australia [25], China [49] and California [41]; *Partitiviridae*-like viruses have been detected in Sweden [45,48], Australia [25], Kenya [49] and California [41]; *Endornaviridae*-like viruses in Australia [25] and *Tymoviridae*-like viruses have been identified in Guadeloupe [46], Kenya [49], California [41], China [53], and Sweden [48]. Moreover, a *Culex Tymoviridae*-like virus (CuTLV) that was isolated from a *Culex* spp. pool from Xinjiang (China) was also shown to produce a cytopathic effect on *Aedes albopictus* C6/36 cell line [55], suggesting a potential plant/mosquito host-shift even when there is no record of mosquitoes as vectors of plant viruses [46]. Nonetheless, there is also the chance that: i) mosquitoes could have acquired these viruses while sap or nectar feeding prior to capture and deposited them on the FTA card along with saliva expectorations as mouthparts contaminants [49,56] while trapped; or ii) they could have been present in the honey used as bait. 

To a lesser extent, the virome profile of FTA cards depicted sequences assigned to three dual-host (mosquito/vertebrate) virus families: *Flaviviridae*, *Phenuiviridae* and *Peribunyaviridae*. *Flaviviridae*-associated sequences were distantly related to two mosquito-specific viruses, Karumba virus (49% amino acid (aa) identity) and Calbertado virus (47–86% aa identities) (Table S2). Reads related to *Phenuiviridae* were assigned to a distant Phasi Charoen-like phasivirus with aa identities ranging from 58% to 77% (Table S2). Meanwhile, most of the *Peribunyaviridae*-associated sequences presented high homologies with Ganda bee virus (35–95% aa identity) (Table 2). Finally, no arboviruses were detected throughout the sampling period by NGS on honey-baited FTA cards. Despite six sequences matched with WNV (59–92% aa identity), these assignations were not taken into consideration due to the length (150 nt), nucleotide identity (<80%) and coverage (<80%) of the sequences.

### 3.3. Viral Genomes Obtained from Honey-Baited FTA Cards

It is noteworthy that de novo assemblies of viral reads from both honey-baited FTA cards batches produced 12 near-complete viral genomes (>98% nucleotide coverage and >93% nucleotide identity) for which the 5′ and 3′ termini are incomplete since RACE-PCRs were not performed. Viral genomes within the orders *Nidovirales* (Alphamesonivirus 1: Ngewotan virus) and *Picornavirales* (e.g., Deformed wing virus and Culex Iflavi-like virus 4), and within unclassified RNA viruses (e.g., Hubei picorna-like virus 61 and Wenzhou soberno-like virus 4) were generated (Table 3). Obtaining near-complete genomes of viruses associated to mosquitoes, highlights the usefulness of FTA cards in preserving viral RNA. However, we cannot exclude that most of the honeybee-related virus genomes might come from the bait. 

### 3.4. Virus Detection by Specific RT-PCRs on Honey-Baited FTA Cards Unexposed to Mosquitoes

To confirm virome results obtained through metagenomics analysis on honey-baited FTA cards, among all the mosquito-associated viruses (Table 2), Alphamesonivirus 1 (3.606.196 abundance in nucleotides), Dezidougou virus (1.424.472 abundance in nts), *Bunyaviridae* environmental sample (335.292 abundance in nts) and Wuhan mosquito virus 7 (43.351 abundance in nts) were selected to design specific primers and set up virus-specific RT-PCRs. All these selected viruses showed to have at least one contig with a matching sequence longer than 1000 nt and similarity above 90%. For identification matters, through the manuscript, these viruses would respectively be referred to as Alphamesonivirus/CAT virus, *Culex* bunyavirus/CAT virus, Dezidougou/CAT virus and Wuhan mosquito/CAT virus. Suffix “CAT” stands for the geographic region of detection, i.e., Catalonia. 

Those honey-baited FTA cards, which were not exposed to mosquitoes recovered from entomological surveys, were then screened individually by virus-specific RT-PCRs to verify the source of the viruses detected by viromics. Screenings of both cards tested negative for *Culex* bunyavirus/CAT virus and Alphamesonivirus/CAT virus, and positive for Dezidougou/CAT virus and Wuhan mosquito/CAT virus. These detections could be explained by (i) the presence of non-culicid dipterans in the traps; they could have deposited these viruses while sugar feeding from the FTA cards, and/or (ii) the source of these viruses came from the honey impregnated on the cards. 

### 3.5. Virus Detection by Specific RT-PCRs on Field-Captured Mosquito Pools

**Figure 2:**
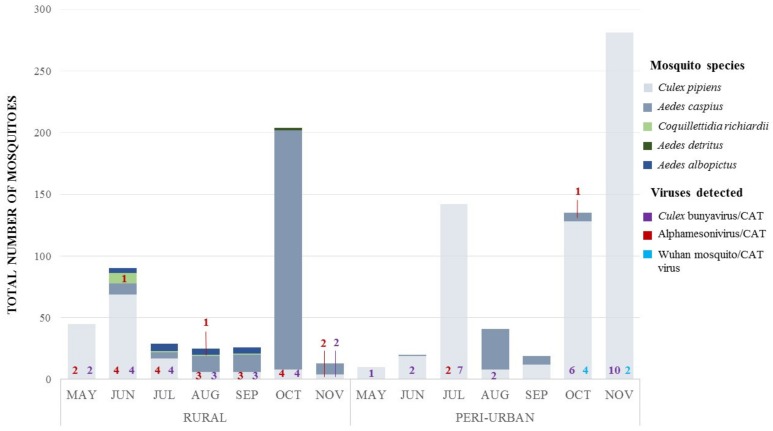
Mosquito species dynamics and virus occurrence in rural and peri-urban biotopes from the Llobregat River Delta. Cumulative bars represent the total number of female mosquitoes captured per month per sampling site. Numbers in color correspond to the total number of mosquito pools that tested positive for a given virus on a particular month and sampling site.

**Figure 3:**
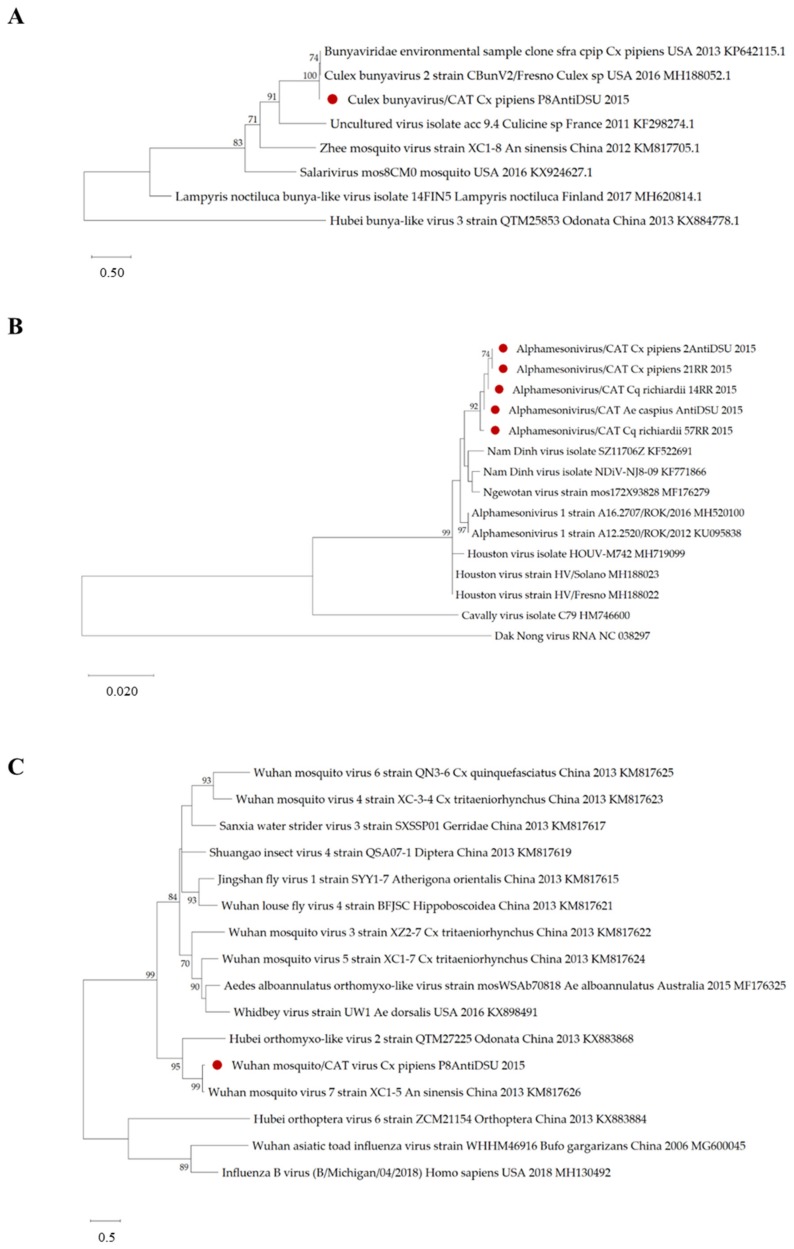
Phylogenetic trees of viruses detected by virus-specific RT-PCR in Catalonian mosquitoes. Trees were drawn to scale, with branch lengths measured in the number of substitutions per site. The percentage of trees in which the associated taxa clustered together is shown next to the branches. Initial tree(s) for the heuristic search were obtained automatically by applying Neighbor-Join and BioNJ algorithms to a matrix of pairwise distances estimated using the Maximum Composite Likelihood (MCL) approach, and then selecting the topology with a superior log likelihood value. Codon positions included were 1st+2nd+3rd+Noncoding. A discrete Gamma distribution was used to model evolutionary rate differences among sites. (**A**) *Culex* bunyavirus/CAT virus, evolutionary history inferred by using the Maximum Likelihood (ML) method and Hasegawa-Kishino-Yano model (HKY+G). The tree with the highest log likelihood (−6117.36) is shown (five categories (+G, parameter = 1.2252)). There were 946 positions in the final dataset. (**B**) Alphamesonivirus/CAT evolutionary history inferred by using the ML method and Tamura-Nei (TN93+G) model. The tree with the highest log likelihood (−2006.57) is shown (five categories (+G, parameter = 0.3417)). There were a total of 839 positions in the final dataset. (**C)** Wuhan mosquito/CAT virus evolutionary history was inferred by using the ML method and Tamura-Nei model (TN93+G). The tree with the highest log likelihood (−8996.79) is shown (five categories (+G, parameter = 0.7704). There were a total of 729 positions in the final dataset.

Virus-specific screenings on mosquito pools confirmed virus circulation as depicted by NGS on FTA cards (Figure 2). Throughout sampling periods, *Culex* bunyavirus/CAT virus (unclassified *Bunyavirales*) was the most common and was recurrently detected in both biotopes (Figure 2). Out of 53 *Cx. pipiens* pools, 50 were found to be infected (including 14 pools unexposed to FTA cards), showing a high occurrence of this viral strain in *Cx. pipiens* mosquitoes from the Llobregat River Delta. BLASTn analysis of the amplified fragment of a RT-PCR positive pool showed a nucleotide similarity of 97.58% to *Bunyaviridae* environmental sample’s RNA-dependent RNA polymerase gene (*RdRp*). Phylogenetically, our strain clustered with *Bunyaviridae* environmental sample (2013) and *Culex* Bunyavirus 2 (2016), which have previously been detected in *Culex* spp. mosquitoes from the United States of America (USA) (Figure 3A). The discovery of *Culex* bunyavirus/CAT virus in Catalonian *Cx. pipiens* widens the range of known distribution for this mosquito-specific bunyaviruses from the USA in California [41,57] and Maryland [42], to Spain. Our findings might also suggest that these bunyaviruses could be genus-specific, as they have been detected only in *Culex* spp. mosquitoes.

Alphamesonivirus/CAT virus, the second most commonly detected virus (Figure 2), was identified in 24 *Cx. pipiens* pools, two *Cq. richiardii* and one *Ae. caspius* pools. *Alphamesonivirus* is the only recognized genus within the mosquito-restricted family *Mesoniviridae* (order *Nidovirales*) [58]. Strains herein reported, shared >98% nucleotide identity to Houston virus and Nam Dinh virus strains’ open reading frame 2 (ORF2) and were closely related to several alphamesonivirus strains that have been detected between 2008 and 2016 in *Culex* spp. mosquitoes. Houston virus (HOUV) and Nam Dinh virus (NDiV) in *Culex quinquefasciatus* from Mexico and China; Ngewotan virus in *Culex australicus* from Australia; NDiV, Alphamesonivirus-1 and HOUV in *Culex* spp. from China, South Korea, and the USA. It is worth mentioning that the viral strains detected in the present study demonstrated a closer relationship to each other than to the strains found in other geographic regions. Moreover, Alphamesonivirus/CAT strains found in *Cx. pipiens,* both rural and peri-urban biotopes, appeared to be more closely related to each other than to those found in other mosquito species from the same geographic area (Figure 3B), thereby suggesting co-evolution events within their host species. These findings, together with the detection of an alphamesonivirus in *Cx. pipiens* from Camargue, France [59], confirm the wide geographical distribution and host range described for the family *Mesoniviridae* [60]. Recently, viruses belonging to this family have been continually detected by virome metagenomics approaches in several mosquito species [41,47,49,53,61], therefore providing more support for this asseveration. 

Finally, Wuhan mosquito/CAT virus was positively detected in six of 53 *Cx. pipiens* pools (Figure 2). Among these, five were captured in traps without honey-baited FTA cards and only one was exposed to a FTA card. Wuhan mosquito/CAT virus exhibited a high phylogenetic relationship (92.15% of nucleotide similarity) with Wuhan mosquito virus 7 strain’s *PB1* gene detected in *Anopheles sinensis* from China in 2013 (Figure 3C). Wuhan mosquito virus 7 belongs to *Quaranjavirus* genus (family *Orthomyxoviridae*, order *Articulavirales*), which has been identified in a pool of *Anopheles sinensis* and *Culex quinquefasciatus* mosquitoes originating from Hubei, China [62]. Finally, throughout screenings, neither *Ae. albopictus* nor *Ae. detritus* were found to be infected by any of those viruses targeted. Detecting *Culex* bunyavirus/CAT and Wuhan mosquito/CAT viruses in *Cx. pipiens* pools, which were not exposed to honey-baited FTA cards, evidenced that these viruses were indeed infecting the mosquitoes and were not acquired while sugar feeding on the FTA cards.

The discovery of *Culex* bunyavirus/CAT, Alphamesonivirus/CAT and Wuhan mosquito/CAT viruses in culicid mosquitoes found in Catalonia, contributes to the knowledge of both the host range and their geographical distribution.

### 3.6. Overall Remarks of the Approach and Future Perspectives

The current study is a pioneer in applying viromics on honey-baited FTA cards during entomological surveys as a tool for the detection of circulating viruses in mosquitoes and the identification of virus in mosquitoes’ saliva. Through this approach, 19 ssRNA (+), six ssRNA (−), eight dsRNA, one ssDNA, five dsDNA viruses and several unclassified viruses were identified; and 12 near-complete viral genomes were obtained from FTA cards, among which seven were linked to mosquito species of sanitary relevance. Acquiring near-complete virus genomes is a clear advantage of metagenomics over classical surveillance based on PCR detection, since insights into the origin, evolution, and diversity of circulating viruses could be gained [25]. Further detection of *Culex* bunyavirus/CAT virus, Alphamesonivirus/CAT virus and Wuhan mosquito/CAT virus in mosquito pools confirmed the presence of these viruses in Europe, where previously their circulation had not been revealed. These findings highlight the value of honey-baited FTA cards combined with viromics in identifying a wide spectrum of viruses that may be associated to sylvan mosquitoes in susceptible areas for arbovirus transmission, without requiring previous knowledge of viral diversity. In future arbovirus surveillance, NGS on honey-baited FTA cards could be used as a guide for prevention and control strategies. In the case of arboviruses detection, entomological surveillance could be exhaustively carried out focusing on specimen classification and molecular analysis where the virus of interest has been previously detected in the FTA cards.

It is worth mentioning that, in spite of the advantages provided by NGS on honey-baited FTA cards, there are some drawbacks that need to be mentioned. Firstly, since FTA cards inactivate the viruses, and NGS provides only genetic information through this approach, no viable virus could be isolated for further characterization. Secondly, virus-bearing mosquito species could not be identified without complementary morphological and molecular analyses. Other possible constraints of this approach could be related to the feeding rate on FTA cards, the quantity of saliva expectorated by mosquitoes, and the number of viral copies liberated within the saliva while sugar feeding. The assumption of blue abdomens in mosquitoes, as the only proof of virus expectoration on FTA cards, might possibly overlook virus release while probing. This fact was evidenced with the detection of chikungunya virus (CHIKV) RNA in FTA cards exposed to experimentally infected *Aedes aegypti* despite the fact that there not been any record of blue dye in their abdomens [16]. Based on these findings, viruses identified by NGS in FTAs could also have been deposited by mosquitoes in which blue abdomen were not present. Furthermore, to improve the sensitivity and efficiency of our approach, honey-baited FTA cards could be placed inside Box gravid traps, as a recent study conducted in Switzerland demonstrated these to be the most effective traps for capturing females of different species when searching for an ovipositional site. In addition, these traps also exhibited the highest feeding success on honey-baited FTA cards [19]. 

The detection of ISVs through metagenomics on honey-baited FTA cards provides evidence that these viruses could be transmitted within mosquitoes’ expectorations, thereby contradicting previous beliefs that they could not be expelled with saliva [19]. Our findings are supported by the tissue tropism evidenced for *Culex* flavivirus (family *Flaviviridae*) and Phasi Charoen-like virus (PCLV) (genus *Phasivirus*, family *Phenuiviridae*), as they were also detected in salivary glands of *Cx. pipiens* from Iowa [63] and in *Ae. aegypti* from South China [64], respectively, and, most importantly, by the detection of *Aedes* flavivirus RNA in saliva from colonized *Ae. albopictus* [65]. Sequences distantly related to PCLV were also detected in our FTA cards. To date, ISVs transmission seemed to be primarily vertical from the adult female to its progeny and venereal from males to females [63,66]. However, horizontal transmission has been hypothesized on breeding sites by direct contact, through feeding in larvae and adults, and/or by copula [40]. Further studies are required to assess the transmission dynamics of the ISVs herein identified.

Furthermore, ISVs are a significant part of the mosquito’s virome. Due to their phylogenetic relationships, great abundance and high diversity, it is presumed that arboviruses might have been originated from arthropod-infecting viruses [67,68,69]. In addition, these viral symbionts are thought to alter the mosquito’s innate immune response, therefore modulating the vector competence for certain arboviruses, and so giving rise to new potential biotools for arbovirus control and prevention [69]. For instance, *Culex* flavivirus naturally infecting *Cx. pipiens* from Colorado possibly suppressed the early infection with West Nile virus (WNV) [70]. In Thailand, Zika virus (ZIKV) and dengue virus 1 (DENV-1) titers in head tissues of *Aedes aegypti* were reduced by intrathoracic inoculation of newly isolated cell fusing agent virus (CFAV) [71]. Likewise, a mosquito flavivirus of natural circulation in *Aedes vexans* form Catalonia seemed to decrease the susceptibility of infection to Rift Valley fever phlebovirus (RVFV) following experimental oral exposure [72]. 

As evidenced, and in spite of the continual discovery of novel mosquito-associated viruses, viral diversity harbored by vector species is still underestimated and little is known about their host range, distribution, ecology and evolution [67,73]. Further studies are required to isolate and fully characterize the genome of Alphamesonivirus/CAT, *Culex* bunyavirus/CAT and Wuhan mosquito/CAT viruses so as to assess their potential as vertebrate pathogens. Finding these ISVs in FTA cards, and therefore in mosquitoes saliva, rises concerns of the potential of these viruses to evolve from being insect-specific to dual-host viruses, acquiring the ability to infect vertebrate cells and become new emerging pathogens. Future surveillance strategies for emerging diseases could include NGS on honey-baited FTA cards to detect previously undiscovered and potentially transmissible viruses so as to prevent new arbovirus outbreaks. 

## 4. Conclusions

The detection of viruses related to *Alphamesonivirus*, *Quaranjavirus* (Wuhan mosquito virus), and unclassified *Bunyavirales* in European field-captured mosquitoes using virus-specific primers derived from metagenomics results, demonstrated that viromics on honey-baited FTA cards is a valid approach for virological surveillance in mosquitoes. To the best of our knowledge, this is the first evidence of circulating ISVs in mosquitoes’ saliva under field conditions. Our study also constitutes the first distribution record of these viruses in the European continent, thereby demonstrating that they are widely distributed despite there being an information gap due to the majority of studies being focused primarily on arbovirus detection. Further studies are needed to better understand the evolutionary history of insect-specific viruses and their potential role in arbovirus transmission. 

## Figures and Tables

**Table 1:** Virus-Specific Primers for RT-PCRs.

Mosquito-Associated Viruses	Primer Code	Primer Nucleotide Sequence (5′→3′)	T*_m_* (°C)	RT-PCR Fragment Size (bp)
Alphamesonivirus 1	ALPMF	GCGCCATTCTGCAGATCAAC	58	1033
	ALPMR	GTGCCAATAAACGCGTGATG		
*Bunyaviridae* environmental sample	BNYF	GAGTCCTTGTCCATCCCYGC	57	1059
	BNYR	GTGCAGGAAGAAGKAGCATGG		
Dezidougou virus	DZGF	GTCCTGTTAAGCTGCAACCC	56	400
	DZGR	CGTAACAACGATAAGTGGCG		
Wuhan mosquito virus 7	WHNF	GCGGAGAGAGGYAAAATGGATC	57	572
	WHNR	CATTCCCATCAGGAACCCTG		

**Table 2:** Mosquito-associated viruses identified in honey-baited Flinders’ Technology Associates (FTA) cards by next-generation sequencing (NGS) analysis. Taxonomic assignations with assembly lengths higher than 400 nt are shown. Abundance and contig length are expressed in nucleotides (nt). Viral identities are expressed in nucleotides and amino acids (aa).

	Closest Hit	Gene/Product	Abundance	aa Identity (%)	Max. Contig Length	% Coverage	nt Identity (%)	Accession No.
Rural	**Alphamesonivirus 1**	**Spike protein, hypothetical protein**	**3606196**	**53–100**	**1328**	**100**	**99.18**	**MF176279.1**
	***Bunyaviridae* environmental sample**	**RNA-dependent RNA polymerase**	**335292**	**49–99**	**4354**	**99**	**99.02**	**KP642114.1**
	*Culex* bunya-like virus	Hypothetical protein	289007	47–100	920	98	98.45	MH188002.1
	*Culex* iflavi-like virus 4	Polyprotein	1009175	71–100	1706	99	95.77	NC_040574.1
	*Culex* picorna-like virus 1	Polyprotein	806998	64–100	1238	100	96.37	MH703059.1
	*Culex*-associated Luteo-like virus	Hypothetical protein, RNA-dependent RNA polymerase	3285	67–100	566	99	95.04	MK440647.1
	Dezidougou virus	Hypothetical protein 1	9366	87–100	638	100	94.34	KY968698.1
	Hubei picorna-like virus 61	Hypothetical protein	53578	84–100	916	99	95.63	KX883915.1
	Wenzhou soberno-like virus 4	Hypothetical proteins 1 and 2	668852	94–98	2284	100	96.67	KX882831.1
	Wuhan mosquito virus 5	PB1	5460	50	580	13	75.95	KX898491.1
Peri-urban	*Aedes pseudoscutellaris* reovirus	VP1	5244	69–100	667	99	78.08	DQ087276.1
	Alphamesonivirus 1	ORF1a, pp1a polyprotein	22590	60–100	932	100	98.18	MH520106.1
	*Culex* Hubei-like virus	Hypothetical protein	5142	85–100	510	91	90.34	MH188025.1
	*Culex* iflavi-like virus 4	Polyprotein	168154	97–100	2170	100	96.04	NC_040574.1
	*Culex* luteo-like virus	RNA-dependent RNA polymerase	16686	42–67	1279	65	67.49	MF176386.1
	*Culex* picorna-like virus 1	Polyprotein	102979	77–100	1290	100	98.29	MH703059.1
	*Culex pipiens* associated Tunisia virus	Replicase	11319	96–100	1446	98	89.11	NC_040723.1
	Culicine-associated Z virus	VP1, RNA-dependent RNA polymerase	14584	77–97	765	96	83.33	KF298283.1
	Daeseongdong virus 1	ORF1, putative RNA-dependent RNA polymerase	614537	75–95	5831	95	82.27	KU095841.1
	**Dezidougou virus**	**Hypothetical protein 1**	**1424472**	**85–100**	**1882**	**100**	**95.42**	**KY968698.1**
	Karumba virus	Similar NS5 protein	96687	49	3160	28	76.31	JF707857.1
	Hubei picorna-like virus 61	Hypothetical protein	5815018	70–100	1252	100	96.01	KX883915.1
	Negevirus nona 1	Hypothetical protein	190830	49–95	2765	99	87.11	AB972669.1
	Wuhan mosquito virus 6	Nucleoprotein	9480	72–100	468	100	97.01	MF176381.1
	**Wuhan mosquito virus 7**	**PB1**	**43351**	**53–100**	**1846**	**100**	**92.15**	**KM817626.1**

Bold type corresponds to the selected viruses for primers design.

**Table 3:** Near-complete viral genomes obtained by NGS on honey-baited FTA cards. Viral assignations with a genome coverage higher than 98% and identities higher than 95% are shown.

Sample	Order	Family	Closest virus	No Reads	Mean coverage per nt	Coverage (%)	% Identity (nt)	Accession No
Rural	*Picornavirales*	*Dicistroviridae*	Kashmir bee virus	28080	401,29 X	100	96.74	AY275710.1
			Black queen cell virus isolate BQCV_MS	3112	49,85 X	100	93.78	MH267694.1
		*Iflaviridae*	Deformed wing virus isolate Hamilton	3921	51,47 X	100	99.77	MF623172.1
			*Culex* iflavi-like 4 virus strain CIVL/Kern	17787	250,75 X	100	95.78	NC_040574.1
	*Nidovirales*	*Mesoniviridae*	Ngewotan virus strain mos172×93828	9326	63,03 X	100	98.88	MF176279.1
		Unclassified RNA viruses	Wenzhou soberno-like virus 4 strain mosZJ35391	12059	562,28 X	99	96.79	KX882831.1
Peri-urban	*Picornavirales*	*Dicistroviridae*	Aphid lethal paralysis virus isolate ALPV-CE	572	8,42 X	99	94.75	JX480861.1
		*Iflaviridae*	Deformed wing virus isolate Hamilton	3670	47,57 X	100	99.75	MF623172.1
			*Culex* iflavi-like 4 virus strain CIVL/Kern	1435	20,74 X	100	95.72	NC_040574.1
		Unclassified RNA viruses	Hubei picorna-like virus 61 strain mosHB235903	147377	2384,82 X	100	95.84	KX883915.1
			Hubei noda-like virus 11 strain arthropodmix22482	210275	6 964,36 X	100	97.58	KX883010.1
			Dezidougou virus strain DEZI/Aedes africanus/SEN/DAK-AR-41524/1984	4939	74,39 X	98	95.32	KY968698.1

## Supplementary Materials

The following are available online at https://www.mdpi.com/1999-4915/12/3/274/s1, Table S1: Species composition and feeding rates on honey-baited FTA cards in peri-urban and rural biotopes from the Llobregat River Delta; Table S2: Viral taxonomic assignations of assembled sequences associated to FTA cards linked to mosquito species during entomological surveys in Catalonia
