# Supplementary material for: Viromics on Honey-Baited FTA Cards as a New Tool for the Detection of Circulating Viruses in Mosquitoes
Source: Viruses. 2020 Feb 29;12(3):274. doi: 10.3390/v12030274 (PMC7150749; doi:10.3390/v12030274)
Supplement: Supplementary file 1 [file viruses-12-00274-s001.pdf]

Supplementary table 1: Species composition and feeding rates on honey-baited FTA cards in peri-urban and rural biotopes from the Llobregat River Delta.

MOSQUITO SPECIES	PERI-URBAN			RURAL			TOTAL
	Total captured	No. of Pools	Blue Abdomen (%)	Total captured	No. of Pools	Blue Abdomen (%)	
Aedes albopictus	0	0	0/0 (0)	20	10	12/20 (60.0)	20
Aedes caspius	48	5	11/48 (22.9)	244	19	41/244 (16.8)	292
Aedes detritus	0	0	0/0 (0)	2	1	0/0 (0)	2
Coquillettidia richiardii	0	0	0/0 (0)	11	5	8/11 (72.7)	11
Culex pipiens	600	31	116/600 (19.3)	155	22	73/155 (47.1)	755

Supplementary table 2: Viral taxonomic assignations of assembled sequences associated to FTA cards linked to mosquito species during entomological surveys in Catalonia. Viral identities are expressed as protein and the taxonomic assignation refers to the virus/common ancestor of the best hits for all the contigs/singletons with the same score. Viral abundance was obtained by summing the length (in nucleotides) of all sequences being associated to this taxon, weighted by the *k-mer* coverage of each contig.

	Order	Family	Genus	Best hit	Peri-urban		Rural		Host
					**% Identity (aa)**	**Abundance (nt)**	**% Identity (aa)**	**Abundance (nt)**	
**ssRNA+**	*Picornavirales*	*Dicistroviridae*	*Aparavirus*	Kashmir bee virus	52-100%	3476802	55-100%	4231521	Hymenoptera
				Israeli acute paralysis virus	-	-	56-95%	123149	Hymenoptera
				Acute bee paralysis virus	69-75%	37594	64-100%	13317	Hymenoptera
				unassigned *Aparavirus*	83-100%	221821	86-100%	378477	
			Triatovirus	Black queen cell virus	46-100%	1612911	63-100%	2224831	Hymenoptera
				Triatoma virus	-	-	52-76%	5666	Hemiptera
			Cripavirus	Aphid lethal paralysis virus	66-100%	104687	67-100%	55235	Hymenoptera
				Rhopalosiphum padi virus	-	-	92-100%	2607	Hemiptera
			Unclassified	Human blood associates *Dicistroviridae*	51-88%	30762	74%	1914	*Homo sapiens*
				Blackberry virus Z	-	-	66-68%	939	Plant
				Bivalve RNA virus G5	38%	23460	-	-	Mollusca
				Formica exsecta virus 1	-	-	75-76%	354	Hymenoptera
				unassigned *Dicistroviridae*	73-100%	17978	80-100%	26396	-
		Iflaviridae	*Iflavirus*	Deformed wing virus	66-100%	2468142	64-100%	1886897	Hymenoptera
				Formica exsecta virus 2	-	-	43%	15081	Hymenoptera
				Sacbrood virus	53-100%	361302	51-100%	593255	Hymenoptera
				unassigned *Iflavirus*	28-100%	739318	72-100%	740894	-
			Unclassified	*Culex* iflavi-like virus 4	97-100%	168154	71-100%	1009175	*Culex* sp.
				Culex iflavi-like virus 3	48-74%	6342	-	-	*Culex* sp.
				Tribolium castaneum iflavirus	40%	42696	28-73%	31272	Coleoptera
				Pityohyphantes rubrofasciatus iflavirus	-	-	55-87%	14170	Arachnida
				other unclassified *Iflaviridae*	70-100%	9183	41-100%	5583	-
		Secoviridae	*Nepovirus*	Red clover nepovirus A	-	-	62%	17857	Plant
				Arabis mosaic virus	61-100%	2294	96-100%	2227	Plant
				Grapevine Bulgarian latent virus	-	-	81-90%	966	Plant

			Grapevine chrome mosaic virus	-	-	43-75%	828	Plant
			unassigned *Nepovirus*	-	-	56-93%	8998	-
		Fabavirus	Prunus virus F	-	-	61%	141	Plant
	Polycipiviridae	unclassified	*Linepithema humile* polycipivirus 2	100%	600	-	-	Hymenoptera
	Picornaviridae	*Enterovirus*	Rhinovirus C	-	-	98-100%	441	*Homo sapiens*
		Sapelovirus	Sapelovirus A	55-100%	579	-	-	Vertebrates
	Unclassified *Picornavirales*		*Culex* picorna-like virus 1	77-100%	102979	64-100%	806998	*Culex* sp.
			Bundaberg bee virus 6	-	-	50-88%	7355	Hymenoptera
			Bundaberg bee virus 2	53-93%	92769	52-96%	6140	Hymenoptera
			Victoria bee virus 1	53-100%	74634	77-100%	6562	Hymenoptera
			Victoria bee virus 2	55-100%	12927	58-100%	6298	Hymenoptera
			Hobart bee virus 1	-	-	48-97%	4653	Hymenoptera
			Darwin bee virus 7	-	-	83-100%	5554	Hymenoptera
			Darwin bee virus 3	44-95%	35457	44-80%	13695	Hymenoptera
			unassigned *Picornavirales*	33-100%	68703	49-100%	1299417	-
Nidovirales	*Mesoniviridae*	*Alphamesonivirus*	Alphamesonivirus 1	60-100%	22590	53-100%	3606196	Mosquitoes
			Alphamesonivirus 2/ Karang Sari virus	-	-	51-86%	4092	*Culex* sp.
			Alphamesonivirus 3/ Dak Nong virus	-	-	70-89%	1770	*Culex* sp.
			Alphamesonivirus 4/ Casuarina virus	-	-	59-100%	2289	*Coquillettidia*
			Alphamesonivirus 5/ Hanalivirus	-	-	55-100%	11157	*Culex* sp.
			Alphamesonivirus 8/ Nse virus	-	-	86%	750	*Culex* sp.
			Alphamesonivirus 9/ Meno virus	-	-	56-81%	7770	*Uranotaenia*
			unassigned *Alphamesonivirus*	69-100%	2118	65-100%	77128	-
		unclassified	Dianke virus	74-100%	22939	-	-	Rodentia
			unassigned *Mesoniviridae*	98-100%	4364	-	-	-
	unclassified *Nidovirales*	-	-	-	-	90-100%	538375	-
Tymovirales	*Tymoviridae*	*Marafivirus*	Grapevine rupestris vein feathering virus	56-88%	53914	77-84%	7359	Plant
			Olive latent virus 3	68%	23922	-	-	Plant
			Citrus sudden death-associated virus	52-83%	55460	-	-	Plant
		Maculavirus	Bee macula-like virus 2	36-73%	266050	-	-	Hymenoptera
			Grapevine red globe virus	41-85%	371176	-	-	Plant
			Culex originated *Tymoviridae*-like virus	40-51%	644699	-	-	*Culex* sp.
			Grapevine fleck virus	53%	13259	-	-	Plant

			Fig fleck-associated virus	48%	7344	-	-	Plant
		Tymovirus	*Erysimum* latent virus	47-76%	74318	-	-	Plant
			unclassified *Tymovirus*	50-88%	1032	-	-	-
		unclassified	Bee macula-like virus	44-61%	9155	-	-	Hymenoptera
			Varroa tymo-like virus	66-68%	462	-	-	Arachnida
			Naranjilla chlorotic mosaic virus	47-67%	10488	-	-	Plant
			unassigned *Tymoviridae*	41-67%	75753	-	-	-
	Alphaflexiviridae	*Potexvirus*	Pepino mosaic virus	69-100%	1957	70-100%	1881	Plant
			White clover mosaic virus	-	-	96-100%	333	Plant
	Betaflexiviridae	*Prunevirus*	*Caucasus prunus* virus	-	-	75-91%	576	Plant
		Carlavirus	Narine latent virus	-	-	70-96%	306	Plant
	unclassified *Tymovirales*		Bat tymo-like virus	41-88%	38435	-	-	Vertebrate
			other unclassified *Tymovirales*	54-94%	756	-	-	-
-	*Flaviviridae*	*Flavivirus*	Quang Binh virus	-	-	55%	135	*Culex* sp.
			Calbertado virus	47-86%	10176	-	-	*Culex* sp.
			West Nile virus	59-92%	672	-	-	Invertebrates/vertebrates
			Meaban virus	72-100%	600	-	-	Invertebrates/vertebrates
		Unclassified	Xishuangbanna *Aedes* flavivirus	-	-	81%	282	*Aedes*
			Karumba virus	49%	96687	-	-	*Anopheles*
			unassigned *Flaviviridae*	-	-	66-83%	204	-
-	*Virgaviridae*	*Hordeivirus*	*Lychnis* ringspot virus	49-97%	46711	45-82%	17047	Plant
		Tobamovirus	unclassified *Tobamovirus*	78-100%	6443	73-98%	19826	-
-	*Narnaviridae*	*Narnavirus*	*Fusarium poae* narnavirus 2	-	-	50-64%	7859	Fungi
			Aspergillus fumigatus narnavirus 2	-	-	68-88%	4930	Fungi
			Aspergillus fumigatus narnavirus 1	-	-	63%	2614	Fungi
		Mitovirus	*Sclerotinia sclerotiorum* mitovirus 27	49-57%	1688	-	-	Fungi
			Rhizoctonia solani mitovirus 6	60-67%	1677	-	-	Fungi
			Erysiphe necator mitovirus 3	58-75%	1599	-	-	Fungi
			Ambrosia artemisiifolia mitovirus 1	45-70%	2482	-	-	Plant
			unclassified *Mitovirus*	-	-	45-87%	1428	-
		unclassified	*Phomopsis longicolla* RNA virus 1	58-74%	396	-	-	Fungi
			other unclassified *Narnaviridae*	48-97%	4724	-	-	-
	-	*Sinaivirus*	Lake Sinai virus 1	90-91%	4183	94-100%	11620	Hymenoptera

				Lake Sinai virus	61-100%	4528	-	-	Hymenoptera
			Ourmiavirus	*Rhizoctonia solani* ourmia-like virus 1 RNA 1	-	-	56-62%	924	Fungi
				Magnaporthe oryzae ourmia-like virus	52-69%	576	-	-	Fungi
			Negevirus	Dezidougou virus	85-100%	1424472	87-100%	9366	*Aedes*
				Negevirus nona 1	49-95%	190830	-	-	*Aedes*
		Luteoviridae	*Polerovirus*	Cucurbit aphid-borne yellows virus	64-98%	735	-	-	Plant
			Unclassified	Culex-associated luteo-like virus	-	-	67-100%	3285	*Culex* sp.
		Potyviridae	*Ipomovirus*	Cucumber vein yellowing virus	-	-	100%	588	Plant
			Potyvirus	Potato virus Y	89-98%	303	-	-	Plant
		Solemoviridae	*Sobemovirus*	Sowbane mosaic virus	-	-	98-100%	444	Plant
			Unclassified	Bat sobemovirus	-	-	60-100%	1917	Vertebrate
		Closteroviridae	*Closterovirus*	Citrus tristeza virus	62-96%	342	75-99%	4164	Plant
		Tombusviridae	*Umbravirus*	*Sclerotinia sclerotiorum* umbra-like virus 3	-	-	63%	2220	Fungi
			Unclassified	*Culex*-associated tombus-like virus	-	-	94-100%	777	*Culex* sp.
				unassigned *Tombusviridae*	65-100%	837	-	-	-
		Bromoviridae	*Ilarvirus*	*Ageratum* latent virus	-	-	90-95%	1269	Plant
				Parietaria mottle virus	-	-	80-93%	1203	Plant
				Fragaria chiloensis latent virus	-	-	63-75%	1143	Plant
				unassigned *Ilarvirus*	-	-	74	292	-
				Tomato necrotic spot virus	80%	6906	-	-	Plant
			Alfamovirus	Alfalfa mosaic virus	-	-	86-98%	405	Plant
			Anulavirus	*Pelargonium zonate* spot virus	39-92%	18444	-	-	Plant
		Nodaviridae	*Alphanodavirus*	Pariacoto virus	51-53%	2238	-	-	Lepidoptera
				unassigned *Alphanodavirus*	-	-	85-87%	1262	-
			Betanodavirus	Wuhan nodavirus	-	-	63%	147	Lepidoptera
			Unclassified	*Culex* mosquito virus 1	-	-	81-100%	1709	*Culex* sp.
				Culex mosquito virus 4	91-100%	3651	-	-	*Culex* sp.
				Culex Hubei-like virus	85-100%	5142	-	-	*Culex* sp.
				other unclassified *Nodaviridae*	50-100%	6063	75-100%	765	-
	Unclassified ssRNA+	-	-	HVAC-associated RNA virus 1	-	-	48-76%	154674	Environmental/ human
				Culex pipiens associated Tunisia virus	96-100%	11319	75-100%	3924	*Culex* sp.
				Chronic bee paralysis virus	-	-	83-100%	7300	Hymenoptera
				other unclassified ssRNA+ viruses	50-100%	5807	44-100%	12887	-
**ssRNA-**	*Bunyavirales*	*Peribunyaviridae*	Unclassified	Seattle Prectang virus	-	-	38-56%	5358	Lepidoptera

				Apis bunyavirus 2	-	-	46-65%	2900	Hymenoptera
				Ganda bee virus	35-95%	174507	60-66%	660	Hymenoptera
			Orthobunyavirus	Buttonwillow virus	-	-	61-69%	294	Culicoides/vertebrate
		Phasmaviridae	*Orthophasmavirus*	Wuchang cockroach virus 1	50%	114	39-80%	13185	Blattodea
			Inshuvirus	Shuangao insect virus 2	-	-	77-91%	1623	Lepidoptera
		Phenuiviridae	*Phasivirus*	Phasi Charoen-like phasivirus	-	-	58-77%	690	*Aedes*
			Phlebovirus	Laurel Lake virus	-	-	54%	373	Arachnida
			Goukovirus	Cumuto virus	-	-	61%	123	*Culex* sp.
			Hudovirus	Hubei lepidoptera virus 1	86%	132	-	-	Lepidoptera
			Unclassified	Citrus concave gum-associated virus	-	-	46%	428	Plant
		Unclassified *Bunyavirales*		*Bunyaviridae* environmental sample	95-98%	294	48-99%	335292	*Culex* sp.
				Culex bunya-like virus	85-100%	1806	47-100%	289007	*Culex* sp.
				Yongsan bunyavirus 1	46%	135	-	-	*Aedes*
				unassigned *Bunyavirales*	-	-	73-100%	213787	-
	Articulavirales	*Orthomyxoviridae*	*Quaranjavirus*	Jingshan fly virus 1	-	-	49-80%	8938	*Muscidae*
				Wuhan mosquito virus 5	-	-	50%	5460	*Culex* sp.
				Wuhan mosquito virus 6	72-100%	9480	45-87%	7813	*Culex* sp.
				Wuhan mosquito virus 7	53-100%	43351	-	-	*Anopheles*
				other unclassified *Quaranjavirus*	-	-	60-100%	618	-
			Unclassified	*Photinus pyralis* orthomyxo-like virus 1	-	-	27%	4445	Coleoptera
				Photinus pyralis orthomyxo-like virus 2	-	-	38%	7165	Coleoptera
	Mononegavirales	*Rhabdoviridae*	*Hapavirus*	Joinjakaka virus	-	-	54-68%	276	*Culicinae* sp.
				Marco virus	54-60%	453	-	-	Vertebrate
			Ephemerovirus	Yata virus	-	-	51%	147	*Mansonia*
				Obodhiang virus	55-72%	414	-	-	*Mansonia*
			Ledantevirus	Keuraliba virus	-	-	65%	123	Rodentia
				Kern Canyon virus	48-52%	309	-	-	Chiroptera/vertebrate
			Sigmavirus	unclassified *Sigmavirus*	61-82%	1107	-	-	-
			Tibrovirus	Beatrice Hill virus	60-76%	693	-	-	Culicoides
			unclassified	unclassified *Rhabdoviridae*	-	-	68-72%	1194	-
				Apis rhabdovirus 1	45-100%	1719	-	-	Hymenoptera
		unclassified	*Anphevirus*	*Aedes* anphevirus	68%	135	68%	144	*Aedes*

		unclassified *Mononegavirales*		unassigned *Mononegavirales*	58-77%	237	-	-	-
	Jingchuvirales	*Chuviridae*	*Mivirus*	Wuhan louse fly virus 6	-	-	56-81%	3844	*Hippoboscidae*
		unclassified ssRNA-		Wuhan louse fly virus 2	-	-	55%	348	*Hippoboscoidea*
				Wuhan louse fly virus 11	61%	126	-	-	*Hippoboscidae*
				other unclassified ssRNA- viruses	-	-	76%	552	-
**dsRNA**		*Partitiviridae*	*Alphapartitivirus*	Pear alphapartitivirus	74-98%	26487	53-98%	299705	Plant
				Medicago sativa alphapartitivirus 1	52-100%	14146	-	-	Plant
				Helicobasidium mompa partitivirus V1-1	-	-	59-86%	67113	Fungi
				unclassified *Alphapartitivirus*	-	-	75-98%	10637	-
			Betapartitivirus	White clover cryptic virus 2	73-100%	2880	82-100%	159048	Plant
				Crimson clover cryptic virus 2	77-100%	3459	56-100%	50325	Plant
				Pleurotus ostreatus virus 1	-	-	61-80%	8364	Fungi
				unclassified *Betapartitivirus*	52-100%	4341	92%	11143	-
			Deltapartitivirus	Spinach deltapartitivirus 1	69-86%	21810	57-92%	6453	Plant
				Pepper cryptic virus 2	53-77%	3828	56-77%	3384	Plant
				Fig cryptic virus	59-84%	9072	63-80%	4075	Plant
				Beet cryptic virus 2	70-84%	4311	64-82%	2193	Plant
				Pepper cryptic virus 1	-	-	60-89%	3693	Plant
				unclassified *Deltapartitivirus*	56-96%	2817	72-77%	2465	-
			Gammapartitivirus	*Fusarium solani* virus 1	61-87%	1107	-	-	Fungi
				Ophiostoma partitivirus 1	-	-	94-96%	357	Fungi
			unclassified	Rose partitivirus	65-97%	7961	55-100%	321156	Plant
				Pittosporum cryptic virus-1	57-96%	5668	-	-	Plant
				Raphanus sativus cryptic virus 3	48-85%	3687	-	-	Plant
				Persimmon cryptic virus	67-79%	3063	-	-	Plant
				Linepithema humile partiti-like virus 1	-	-	69-100%	195870	Hymenoptera
				Podosphaera prunicola partitivirus 4	-	-	44-78%	25768	Fungi
				other unclassified *Partitiviridae*	46-100%	13507	49-100%	79693	-
		Totiviridae	*Totivirus*	*Saccharomyces cerevisiae* virus L-A-lus	-	-	47-90%	887016	Fungi
				Saccharomyces cerevisiae virus L-A	49-87%	8727	-	-	Fungi
				Scheffersomyces segobiensis virus L	51-92%	2439	36-88%	42633	Fungi
				unclassified *Totivirus*	52-89%	1191	49-68%	3183	-
			unclassified	*Pterostylis sanguinea* totivirus A	54-95%	3489	66-97%	268125	Plant
				Saccharomyces kudriavzevii virus L-A-1082	64-96%	3120	46-100%	45147	Fungi

			Saccharomyces paradoxus virus L-A-4650	-	-	53-56%	25000	Fungi
			Saccharomyces paradoxus virus L-A-45	42-89%	11412	47-85%	9434	Fungi
			Saccharomyces kudriavzevii virus L-A1	-	-	60-74%	7425	Fungi
			Saccharomyces uvarum virus L-A-10560	-	-	55-78%	3957	Fungi
			Camponotus nipponicus virus	29%	11361	-	-	Hymenoptera
			*Camponotus yamaoka*i virus	56%	3780	-	-	Hymenoptera
			other unclassified *Totiviridae*	41-97%	19816	55-71%	19494	-
	Endornaviridae	*Alphaendornavirus*	*Phaseolus vulgaris* alphaendornavirus 2	42-73%	13314	38-78%	10353	Plant
			Helianthus annuus alphaendornavirus	54%	3996	-	-	Plant
			Persea americana alphaendornavirus 1	-	-	54-100%	16536	Plant
			Bell pepper alphaendornavirus	41-100%	3693	-	-	Plant
			Grapevine endophyte alphaendornavirus	68-100%	3648	-	-	Plant
			Winged bean alphaendornavirus 1	36-90%	7604	46-88%	4077	Plant
			unclassified *Alphaendornavirus*	34-100%	7899	42-100%	7716	-
		unclassified	*Phaseolus vulgaris* endornavirus 3	64-87%	2377	62-83%	3714	Plant
			Ceratobasidium endornavirus G	43%	2133	-	-	Fungi
			Rhizoctonia solani endornavirus 1	60%	1881	-	-	Fungi
			other unclassified *Endornaviridae*	47-95%	1125	-	-	-
	Picobirnaviridae	unclassified	unclassified *Picobirnaviridae*	60-87%	2550	48-100%	15021	-
	Chrysoviridae	*Chrysovirus*	*Persea americana* chrysovirus	86-100%	1023	57-100%	1607	Plant
	Hypoviridae	unclassified	*Sclerotinia sclerotiorum* hypovirus 3	-	-	76-100%	1170	Fungi
			unclassified *Hypoviridae*	92-100%	597	-	-	-
	Amalgaviridae	unclassified	Rubber dandelion latent virus 2	-	-	61-63%	354	Plant
			unclassified *Amalgaviridae*	63-86%	468	-	-	-
	Reoviridae	*Phytoreovirus*	Rice gall dwarf virus	66-67%	273	56-100%	1035	*Cecidomyiidae*
		Dinovernavirus	Fako virus	61-100%	19596	56-83%	366	Mosquito
			Aedes pseudoscutellaris reovirus	69-100%	5244	-	-	
		Fijivirus	unclassified *Fijivirus*	59-66%	432	-	-	-
		unclassified	Soudat virus	76-100%	2093	67-100%	1641	*Drosophilidae*
			Cimodo virus	98-100%	372	70-100%	1008	*Culicidae*
			Grange virus	55-59%	396	-	-	*Drosophilidae*
			other unclassified *Reoviridae*	-	-	53-66%	795	-
unclassified dsRNA viruses			*Rosellinia necatrix* fusagravirus 3	-	-	41%	49392	Fungi
			Colletotrichum acutatum RNA virus 1	-	-	75-92%	1653	Fungi

**Unclassified RNA viruses**				Hubei partiti-like virus 34	68-100%	2214913	55-100%	1997449	Gastropoda
				Wenzhou sobemo-like virus 4	-	-	94-98%	668852	Mosquitoes
				Hubei mosquito virus 2	-	-	37-85%	119667	Arthropoda
				Hubei picorna-like virus 61	70-100%	5815018	84-100%	53578	Mosquitoes
				Hubei picorna-like virus 57	-	-	72-100%	45296	Diptera
				Hubei picorna-like virus 15	-	-	58-100%	40767	Arthropoda
				Hubei picorna-like virus 63	55-100%	986925	-	-	Insecta
				Hubei noda-like virus 11	55-100%	749763	-	-	Arthropoda
				Hubei picorna-like virus 62	68-100%	432814	-	-	Arachnida
				Beihai tombus-like virus 11	41-71%	118730	-	-	Actiniaria
				Hubei picorna-like virus 60	38-75%	144477	-	-	Arthropoda
				Hubei macula-like virus 2	58-70%	116307	-	-	Odonata
				other unclassified RNA viruses	34-100%	414971	40-100%	106752	-
**ssDNA viruses**	-	*Parvoviridae*	*Ambidensovirus*	Hymenopteran ambidensovirus 1	51-60%	850	-	-	Hymenoptera
				Lepidopteran ambidensovirus 1	51%	522	-	-	Lepidoptera
				unassigned *Ambidensovirus*	58-75%	642	-	-	-
**dsDNA viruses**	*Caudovirales*	*Siphoviridae*	*Sextaecvirus*	*Staphylococcus* phage 6ec	38%	126	-	-	Bacteria
		unclassified *Caudovirales*		*Rhizobium* phage RHEph10	40%	5827	-	-	Fungi
	-	*Poxviridae*	*Alphaentomopoxvirus*	*Anomala cuprea* entomopoxvirus	31%	2313	-	-	Coleoptera
			Betaentomopoxvirus	*Adoxophyes honmai* entomopoxvirus 'L'	43-73%	804	-	-	Lepidoptera
	-	*Baculoviridae*	*Alphabaculovirus*	*Lymantria xylina* nucleopolyhedrovirus	-	-	60%	1107	Lepidoptera
		Hytrosaviridae	*Muscavirus*	*Musca domestica* salivary gland hypertrophy virus	-	-	69%	706	Muscidae
		Nudiviridae	unclassified	Tomelloso virus	-	-	64-70%	531	*Drosophilidae*
		unclassified dsDNA viruses		*Apis mellifera* filamentous virus	55-100%	452461	54-100%	617009	Hymenoptera
**Unclassified viruses**				Daeseongdong virus 1	75-95%	614537	-	-	*Culex* sp.
				Dansoman virus	-	-	44%	4110	*Drosophilidae*
				Tomato matilda virus	88-100%	51998	-	-	Plant
				Diabrotica virgifera virgifera virus 1	41-66%	19503	-	-	Coleoptera
				Culicine-associated Z virus	77-97%	14584	-	-	*Culicinae* sp.

Araticum virus	70-85%	25767	69-92%	10435	Plant
Bloomfield virus	52-100%	7584	46-100%	16825	*Drosophilidae*
Linepithema humile virus 1	-	-	25-100%	6134	Hymenoptera
Basavirus sp.	-	-	44-55%	5979	Vertebrate
Culex luteo-like virus	42-67%	16686	-	-	*Culex* sp.
other unclassified viruses	50-100%	6872	-	-	-
